# Supplementary material for: Are carbon emissions trading and green financial instruments synergistic? -Comprehensive quantitative research based on content analysis
Source: PLoS One. 2024 Mar 7;19(3):e0298601. doi: 10.1371/journal.pone.0298601 (PMC10919601; doi:10.1371/journal.pone.0298601)
Supplement: S1 Data — (ZIP) [file pone.0298601.s001.zip › Data/地级市Z_F工作报告.zip!/║╙─╧/╨φ▓2╩╨/└┤╫╘╣1⁄2╓┌║┼í╛┬φ┐╦╩2╛▌═°í┐_2008.docx]

各位代表：

现在，我代表市人民政府，向大会作工作报告，请予审议，并请市政协各位委员提出意见。

一、2007年工作回顾

2007年是本届政府任期的第一年。市人民政府在省委、省政府和市委的正确领导下，以邓小平理论和“三个代表”重要思想为指导，全面贯彻党的十七大精神，认真落实科学发展观，积极构建和谐社会，求真务实，开拓创新，较好地完成了市五届人大一次会议确定的各项任务。全市生产总值达到867.25亿元，增长14.4%；工业增加值517.59亿元，增长17.7%；全社会固定资产投资397.22亿元，增长35.3%，其中城镇以上固定资产投资318.11亿元，增长38.9%；全年社会消费品零售总额200.75亿元，增长18.3%；进出口总额7.74亿美元，增长35.2%，其中出口总额6.14亿美元，增长34.1%；地方财政一般预算收入33.76亿元，增长32.1%；城镇居民人均可支配收入10741元，实际增长15.6%；农民人均纯收入5046元，实际增长12.1%；城乡居民储蓄存款余额319.06亿元，增长7.6%；人口自然增长率控制在4.45‰以内；城镇登记失业率控制在3.33%以内；万元生产总值能耗降低4%。全市综合实力跨上新台阶，结构调整取得新成效，城乡面貌又有新变化，生态环境得到新改善，人民生活有了新提高，经济社会保持了又好又快发展的良好态势。

（一）注重在加快项目建设中夯实发展基础。我们坚持把项目建设作为加快经济社会发展的重要支撑，着力优化投资结构。抓住事关全市长远发展的187个重点项目，累计完成投资141.5亿元，超年度计划11.5亿元，同比增长45%，是近几年来进度最快、成效最好的一年。东城区热电厂建成试运行；泉店煤矿建设完成总投资的68.3%；烟草薄片项目即将竣工投产；禹州电厂二期工程获国家发改委核准，全面开工；许昌铁矿开工建设。禹登铁路主体工程基本完工，许扶高速、禹登高速、郑石高速许昌段和许禹高速公路竣工通车。许昌职业技术学院、许昌市二高迁建一期工程竣工投用。许昌市残疾人康复中心、许昌群众艺术馆主体工程全部完工。许昌社会福利院建设进展顺利。万通汽贸城、河南冷王物流园、河南众品低温物流中心等现代物流业项目竣工运营。一批重大项目的建成为许昌经济社会发展奠定了良好的基础。

（二）注重在优化经济结构中转变发展方式。我们以新型工业化为核心，大力发展集群经济，持续推进产业结构调整。规模以上工业增加值完成355.5亿元，增长22.2%，利润总额162.12亿元，增长68.3%，产业竞争力不断提升。着力推动“一带九区二十个产业集群”建设，项目入驻率达到70%，集约化水平明显提高。实施“千百亿”工程，40户重点企业、100户“小巨人”企业、20户重点高新技术企业成为新的经济增长点，企业组织结构明显优化。加快发展商贸物流、文化旅游等现代服务业，第三产业增加值194.26亿元，增长14.3%，达到近年来最高水平。扎实开展“品牌兴市”活动，大力实施标准化战略，创中国名牌产品6个、中国驰名商标2个、国家免检产品2个。组织实施省级以上科技项目58项，2家企业被确定为国家级创新型试点企业，高新技术产业增加值占工业增加值的比重达到22.3%，科技进步对经济增长的贡献率达到51%，连续3次被评为全国科技进步先进市。严格落实节能减排，机立窑小水泥和25台小火电机组全部关停，509座黏土砖瓦窑场全部拆除，化学需氧量和二氧化硫排放量分别削减4007吨和5834吨。全市经济在又好又快发展进程中迈出了坚实的步伐。

（三）注重在破解“三农”问题中扎实建设新农村。我们坚持统筹城乡发展，努力促进农业增效、农民增收和农村发展。粮食生产持续增长，总产达到273.4万吨，再创历史新高。种粮直补、农资综合直补、良种补贴、农机具购置补贴等各项惠农政策全面落实，累计发放补贴资金2.4亿元。种植业结构不断优化，花卉苗木、蔬菜、烟叶和中药材种植面积分别达到61万亩、99万亩、21.5万亩和40万亩。农业产业化经营健康发展，新增省级农业产业化龙头企业18家。现代畜牧业加快发展，总产值95亿元，占农业总产值的43%。以“饮干净水、走平坦路、建沼气池、用卫生厕”为重点，不断完善农村基础设施，新建农村沼气池5.19万口，新解决13.5万人的饮水安全问题，农村公路完成建设里程914.8公里，村村通全部实现，村互通加快推进。城乡一体化推进区建设取得显著成效，编制完成了总体规划和30个专业规划，24条道路和其他配套设施开工建设，许昌至长葛城际快速通道建成并开通公交，一批工业项目、生态绿化和村庄改造建设启动实施，文化、教育、体育、卫生、社会保障在推进区协调推进、健康发展，呈现出勃勃生机和良好的发展态势。推进区在全市统筹城乡发展的实践中发挥了示范带动作用。

（四）注重在提高城市建管水平中加快推进城镇化。我们努力创新城市发展理念，不断提高城镇综合承载能力，全市城镇化率达到35.79%，较上年提高1.88个百分点。按照《许昌市城市总体规划》，改造提升老城区，建设完善东城区，有序开发推进区。开工建设41个城建重点项目，完成投资6.87亿元，许都公园、帝豪游园、魏武帝广场、许扶运河公园建成开放，107国道城区段改线、京珠高速南连接线改造、延安路南北贯通、万丰路综合整治等城市道路竣工通车。市区积水点改造工程顺利完成。集中整治市区运粮河等5条内河，基本实现了清水长流。城市空气质量优良天数达到338天。城区精细化管理和亮化工程成效显著，城市品位和形象明显提升。“国家森林城市”实现夺杯拿牌，“国家卫生城市”通过暗访考核，被命名为第一批省级文明城市。县域经济综合实力进一步增强，生产总值达到773.2亿元，增长16.2%，长葛市、禹州市在全省20强中的位次再次前移。县（市）城区和中心镇建设步伐加快，公共设施和社会服务日趋完善，布局合理、城乡协调的城镇化发展新格局初步形成。

（五）注重在保障和改善民生中构建和谐许昌。我们坚持让改革发展的成果惠及人民，集中人力和财力解决涉及群众切身利益的实际问题，市委、市政府承诺的“十件实事”全部完成。财政一般预算支出中涉及民生的支出29.4亿元，占总支出的46.3%，增长35.8%。投入1.54亿元，免除农村义务教育阶段中小学生学杂费、书本费和补助贫困生生活费，惠及学生50.73万人；投入2437万元，为874所农村贫困地区中小学校更新15.2万套课桌凳；维修改造农村中小学校舍5.9万平方米，超额完成年度计划。投入3134.9万元，新型农村合作医疗实现全覆盖，平均参合率达到87.6%；21所国债项目乡镇卫生院投入使用。不断扩大就业与社会保障覆盖面，新增城镇就业62283人，新增城镇基本养老保险13673人，新增城镇基本医疗保险37939人。全面完成农村低保提标扩面任务，农村低保对象补助标准提高到每人每月30元。89个敬老院建成并投入使用，五保户集中供养率提高到31%。廉租住房制度全面落实，市区558户符合条件的住户实现了应保尽保。努力增加城乡居民特别是低收入居民收入，提高了全市最低工资标准和企业退休人员基本养老金发放标准，全面推进机关事业单位工资收入分配制度改革，规范了公务员津贴补贴，人民群众得到了更多实惠，生活水平普遍提高。

（六）注重在推进改革开放中增强发展活力。我们着力推进企事业单位改革改制，全市完成68家。许继集团改制和引进战略投资者取得实质性进展，美菊鞋业、豫中纺织等改制企业的遗留问题得到妥善解决。加快推进企业上市，9家企业先后启动了上市准备工作。围绕“审批瘦身、运作阳光、服务提质”，新取消行政审批项目32项、行政事业性收费项目113项，清理罚款项目329项。开通网上行政审批系统，85项行政审批项目实现在线处理。新的行政服务大厅落成使用，市级招标投标交易中心投入运行。“效能提升年”活动深入开展，84个市直单位实行了标准化管理，机关作风明显改善。出台实行了财政投资评审办法，审减投资额7206万元，审减率23.7%。全面推行土地招拍挂制度，打造阳光土地市场，土地出让更加规范透明。监察、审计等专门监督工作进一步改善和加强。对外开放迈出新步伐，新批准设立外商投资企业18家，实际利用外资8817万美元，同比增长35.5%。

（七）注重在加强精神文明和法制建设中促进社会大局稳定。我们切实保障人民权益，维护社会公平正义。深入开展“五五”普法和“讲正气、树新风、促效能”主题教育活动，精神文明和民主法制建设取得新成绩，获得国家级“民主法治示范村”3个、省级文明单位28个。坚持依法行政，全面推行政务、村务、厂务公开。自觉接受人大法律监督和政协民主监督，办理人大代表议案、建议72件、政协委员提案242件，办复率100%。查处商业贿赂案件43起，教育乱收费、医药购销和医疗服务中的不正之风、公路“三乱”等损害群众利益的突出问题得到有效解决，从源头上预防和治理腐败工作扎实开展，廉政建设取得新的成效。加强社会治安综合治理，依法严厉打击各种违法犯罪活动，人民群众的安全感进一步增强。重视人民群众来信来访，设立了市长信箱、网上信访等多种反映群众诉求的渠道。推行电子政务，完善新闻发布会制度，提高了政府工作效率和透明度。完善110城市应急联动服务系统和应急救援联动机制，处理公共突发事件的能力进一步增强。军政军民团结不断加强，民族宗教、外事侨务、地方史志、档案、地震、气象等工作都取得了新的进展。

各位代表，一年来的成就，是省委、省政府和市委正确领导的结果，是市人大、市政协监督、支持的结果，是全市人民扎实工作、团结实干的结果。值此，我代表市人民政府，向全市人民和各界人士，向所有关心、支持许昌发展的同志们、朋友们表示衷心的感谢！

在看到成绩的同时，我们也清醒地认识到，当前经济运行中存在的价格持续上涨、资源环境约束加剧、节能减排任务加重、安全生产形势依然严峻等矛盾和问题，对我们实现又好又快发展带来了一定困难；国家实行从紧的货币政策，严把土地闸门，提高市场准入门槛，对正处于工业化、城镇化加速阶段的许昌带来了新的挑战；基础条件差、经济总量小、产业层次低、城镇化水平低、经济开放度低等深层次问题的存在，对我们实现跨越发展带来了较大压力；社会管理和公共服务比较薄弱、个别部门依法行政观念淡薄、办事效率低下、损害群众利益的行为时有发生、腐败现象不同程度地存在，对政府的自身建设带来了新的考验。我们将始终保持清醒头脑，切实增强忧患意识，正视问题，知难而进，扎实工作，绝不辜负全市人民的重托。

二、今年工作的总体要求和奋斗目标

今年是全面贯彻落实党的十七大精神的第一年，做好今年的工作意义重大。今年政府工作的总体要求是：以党的十七大精神为指导，以市委五届五次全会确定的工作任务为目标，全面贯彻落实科学发展观，着力转变发展方式，更加注重结构调整和提高发展质量，更加注重推进改革开放和自主创新，更加注重节能减排和生态环境保护，更加注重改善民生和促进社会和谐，推动经济社会又好又快发展，为实现富民强市目标而奋斗！

今年全市经济社会发展主要预期目标是：生产总值增长13%；财政一般预算收入增长19%；工业增加值增长16%；全社会固定资产投资增长21%，其中城镇固定资产投资增长26%；社会消费品零售总额增长15%；进出口总额增长17%，出口总额增长18%；城镇居民可支配收入增长12%，农民人均纯收入增长8%；人口自然增长率控制在5‰以内；新增城镇就业5.5万人，城镇登记失业率控制在4%以内；单位生产总值能耗下降4.13%，化学需氧量控制在1.38万吨以内，二氧化硫排放量控制在3.66万吨以内。

面对科学发展的新任务、新要求，我们必须不断解放思想、开阔视野，审时度势、把握规律，全力以赴、推动发展。做好今年政府工作必须把握好以下4个原则：第一，必须树立发展的新理念，着力在统筹兼顾中推动科学发展。坚持以人为本，坚持统筹发展，促进当前与长远、城市与乡村、经济社会与生态环境的全面协调可持续发展，不断寻找新机遇，不断培育新优势，以科学的方法和实绩践行科学发展观。第二，必须突出发展的新重点，着力在改善民生中促进社会和谐。准确把握人民群众的所需、所急、所忧、所盼，更加注重社会建设，致力促进全民创业，切实保障和改善民生，让改革发展成果更好地普惠于广大群众，特惠于困难群体。第三，必须建立发展的新机制，着力在推进改革开放中增添发展活力。坚定不移地深化改革，扩大开放，苦干实干，以思想观念的解放促进经济社会的发展，以改革创新的精神破解发展的瓶颈，以求真务实的作风推进工作的落实。第四，必须适应发展的新要求，着力在加强政府自身建设中提高服务水平。坚持把实现好、维护好、发展好最广大人民的根本利益，作为政府工作的出发点和落脚点，推进环境创优，提升行政效能，为人民服务，做人民公仆。

三、坚持科学发展，努力转变经济发展方式

坚持产业立市，大力推进经济结构战略性调整，促进一、二、三产业协调发展，实现速度、质量、效益有机统一。

（一）加快重点项目建设，促进支柱产业发展

依靠项目承载投资、促进创业、改善民生、加快发展。突出抓好总投资435亿元的110个重点建设项目，力争当年完成投资168亿元。着力推进禹州电厂二期、许昌铁矿、南水北调许昌段、京珠高速公路拓宽许昌段等一批投资10亿元以上的重大项目建设。加快农业、水利、交通、能源、社会事业等基础产业和基础设施建设，统筹推进教育、卫生、文化和其他涉及民生的社会事业项目。扎实推进能源电力、卷烟、电器、煤化工等重大工业前期项目。继续完善和落实重点项目建设管理的各项制度，为重点项目建设提供良好的环境。

（二）大力发展集群经济，走新型工业化道路

以新型工业化为核心，推进结构调整，培育特色产业，大力发展集群经济。扎实推进“千百亿工程”，积极推动电气制造、能源电力、有色金属、汽车零部件等行业的重点企业进行战略重组，争取1至2家企业销售收入超百亿元。继续扶持40户重点企业、100户“小巨人”企业和20户重点高新技术企业加快发展，不断优化企业组织结构。认真落实《许昌—长葛集群经济产业带发展规划》，着力打造“一带九区二十个产业集群”，切实提升集约化水平。加强对工业运行的监测，提高运行质量，防范和化解企业风险。加强自主创新，积极推动企业技术中心、工程研究中心等科技创新体系建设，力争科技对经济增长的贡献率达到52%以上。继续实施“品牌兴市”战略，积极创建国家级、省级名牌产品和区域品牌，努力形成一批具有地方特色的品牌产品、品牌企业和品牌集群。

（三）统筹城乡发展，扎实建设社会主义新农村

按照城乡经济社会发展一体化新格局的要求，积极促进农业稳定发展、农民持续增收，切实解决农村民生问题。认真落实各项强农惠农政策，抓好粮食生产，总产量稳定在260万吨。以特色农业、高效农业、生态农业、有机农业、循环农业为抓手，着力提升现代农业发展水平。大力发展花卉苗木、中药材、蔬菜、烟草等特色产业，建设标准化生产基地，花卉苗木种植面积达到75万亩。重点抓好106家市级以上农业龙头企业，推进农业产业化经营。加快建设畜牧业“千场百区”和“一园三基地”，促进现代畜牧业发展。加强农村基础设施建设，扎实开展村庄分类整治，着力改善村容村貌。改造县乡公路200公里、通村公路180公里，解决15万人的饮水安全问题，新建农村户用沼气6万口，绿化村（镇）715个。坚持严格的耕地保护制度，加大土地开发整理复垦力度，坚决制止违法违规占用耕地行为。实施农业综合开发，搞好“农田机井通电”工程建设，改造中低产田7万亩，新增有效灌溉面积5万亩。完成病险水库除险加固任务。强化以公益性为主的多元化农业技术推广服务，推进农业科技创新和成果转化。按照“六个一体、三个集中”的要求，全面加快城乡一体化推进区建设步伐。着力抓好142个建设项目，完善基础设施，强化产业支撑，突出生态特色。高标准建设禄马村等“五朵金花”，推进有条件的村庄逐步建成农村新社区。大力发展教育、医疗、卫生、社会保障等社会事业，推进户籍改革。鼓励、支持禹州、襄城、鄢陵等县（市）探索统筹城乡发展的新路子。

（四）着力发展服务业，推进经济结构调整

加快发展服务业，是转变经济发展方式的必由之路。要以发展商贸物流业、文化旅游业、社区服务业和农村服务业为重点，不断提升服务业的比重和水平。着力培育20户服务业重点企业，积极推进丹尼斯广场、胖东来国际商城、万里物流园区、许昌汽车零部件市场等大型零售商业和仓储物流业项目建设。大力推进灞陵桥景区综合开发、曹魏三国园等16个文化旅游产业重点项目建设，扎实做好灵井“许昌人”遗址的宣传、保护、规划、利用工作，不断培育旅游精品线路和景区。加快发展社区服务业，积极发展信息、金融保险、证券等现代服务业，促进房地产业规范、持续、健康发展。扎实推进家电下乡试点工作，激活农村消费市场。继续抓好“万村千乡市场工程”，新增农家店300家，覆盖率达到85%，逐步健全农村市场体系。

（五）增强中心城市带动能力，加快推进城镇化

按照带状城市发展规划，适度拉大城市框架，着力提升城市内涵，逐步完善整体功能，切实发挥辐射带动作用，不断提高城镇综合承载能力，城镇化率提高1.5个百分点。坚持规划先行，科学编制、依法实施各类城镇规划，着力构筑符合实际的城镇化发展体系。切实抓好城区道路桥梁、老城区和城中村改造、环卫设施、人行道改造、管网入地、农贸市场改造、市区畅通工程等10大类55个城建重点项目建设，完成投资14.6亿元。继续深化“四城同创”工作，确保实现创建国家卫生城市夺杯拿牌，创建国家环保模范城市全面达标，创建国家森林城市巩固提升，创建国家文明城市扎实推进。坚持建管并重，积极推进城市管理精细化，探索管理手段数字化，打造城市夜景灯饰化，全面提升城市管理水平。加快发展县（市）城区，支持有产业支撑的乡镇合理规划、突出特色，建设一批中心镇。继续把发展县域经济作为提升区域竞争力的重要途径，通过强化工业主导、发展集群经济、加快项目建设、壮大非公经济、统筹城乡发展、提升现代农业，促进县域经济又好又快发展。

（六）落实节能减排，促进经济社会可持续发展

本着对人民负责、对子孙后代负责的态度，切实抓好节约资源和环境保护。实行严格的问责制和“一票否决”制，确保完成节能减排目标任务。关停不符合产业政策的焦化生产企业，淘汰落后产能60万吨。继续做好内河整治，加大执法力度，坚决杜绝企业偷排和超标排污现象。继续推进瑞贝卡污水净化二期工程、许昌县尚集污水处理厂、长葛市城南污水处理厂、长葛市白寨制革园和一林纸业深度治理工程等项目，确保早日建成投用。加快中水回用工程和污水配套管网建设，不断提高污水处理能力。切实做好第一次污染源普查工作。严格市场准入，对达不到合理用能标准和节能规范要求的新建项目，不审批、不供地、不放贷。大力发展循环经济，全面完成9个工业集聚区循环经济发展规划的制定，积极推进大周镇再生金属回收加工区和襄城县煤化工循环工业聚集区建设。全面落实林业生态建设规划，力争提前2年实现林业生态许昌的建设目标。让许昌水更清、天更蓝、城更绿，让人民群众的生活环境更加美好。

四、坚持改善民生，致力建设和谐许昌

顺应人民群众过上更好生活的新期待，是我们一切工作的出发点和归宿。要带着责任和感情更好地改善人民生活，让全市人民生活得更踏实、更幸福。

（一）注重社会建设，努力保障和改善民生

继续从解决人民群众最关心、最直接、最现实的利益入手，着力办好2008年“十大实事”。开工建设市综合博物馆、职业技术学院体育馆，加快推进市文化会展中心、社会福利院、东城区中小学等社会事业项目建设。优先发展教育，继续增加农村义务教育公用经费，提高保障水平。适当提高农村家庭经济困难寄宿生生活费补助标准。改造、新建农村中小学校舍11.7万平方米，维修改造学校147所。认真落实保障经济困难家庭、进城务工人员子女平等接受义务教育政策。从今年秋季起全面免除城市义务教育学杂费，在全市城乡普遍实行免费义务教育。继续做好市区中小学布局调整。鼓励和规范民办教育发展，大力发展职业教育，积极推进职业技术学院二期建设。完善公共卫生服务体系，建成24个社区卫生服务中心，着力提高78所乡镇卫生院的技术、服务和管理水平，改造农村厕所2400座。发展文化、体育、广播电视事业，繁荣群众文化生活。继续推进农村电影公益放映工程和文化信息资源共享工程。做好广播电视“村村通”工作，年底前实现市级广播电视节目全覆盖。新增农村有线电视2万户，在推进区建制村实现全覆盖。继续推进体育器材进社区，加快农民体育健身工程建设，办好市第六届运动会。认真落实计划生育政策，持续稳定低生育水平。

（二）打造最佳创业基地，以创业带动就业

把就业作为民生之本，以创业在许昌为主题，全面实施“创业富民、就业惠民”工程。加大就业援助力度，实现下岗失业人员再就业1.1万人，动态消除“零就业家庭”。加强农村劳动力就业技能培训，完成培训12.8万人，新增输出转移就业8.5万人。鼓励引导大中专毕业生、外出务工人员回乡创业。大力发展非公经济，提升非公经济比重和水平，促进多种所有制经济共同发展，激发全民创业活力。强化创业政策扶持，加大税费优惠和信贷支持力度，在市、县两级建立创业项目库，推进创业孵化园区和创业示范基地建设，力争用２至3年时间把推进区建设成为全民创业的示范区，使许昌成为干事创业、投资兴业的热土。

（三）完善社会保障体系，保障人民基本生活

建立完善覆盖城乡的社会保障体系，消除人民生活的后顾之忧。加快城乡居民医疗保障制度建设，新增参保城镇职工基本医疗保险1万人；积极开展城镇居民基本医疗保险试点，参保人数覆盖50%以上；全面推行新型农村合作医疗制度，参合标准由每人每年50元提高到100元，参合率达到90%以上。继续提高企业退休职工基本养老金水平，人均增加100元以上，新增参保城镇职工基本养老保险1.5万人。高风险企业农民工基本参加工伤保险。建立城乡低保补助标准自然增长机制，城市低保人均月补差标准由87元提高到95元，农村低保标准由每月30元提高到40元，实现动态管理下的应保尽保。新建、改扩建农村敬老院46个，新增集中供养五保对象3700人，五保户集中供养率达到45%。全市新建廉租房1.6万平方米、经济适用住房18万平方米，落实城市低收入住房困难家庭住房补贴制度。全面完成省属煤炭企业棚户区954户困难群众住房改造任务。落实好稳定物价的各项政策措施，加强监管，保持市场物价基本稳定。按照政策，全面推进机关事业单位工资收入分配制度改革，落实职工最低工资标准，使人民群众得到更多实惠。

（四）加强精神文明和民主法制建设，维护社会大局稳定

大力加强社会主义精神文明建设，强化公民的社会主义核心价值体系教育，提炼许昌精神，倡树文明新风。深入推进依法治市工作，积极开展法律援助，维护公平正义。切实加强信访和人民调解工作，深入开展矛盾纠纷的排查和处理，努力化解社会不稳定因素。严格落实安全生产责任制，继续开展煤矿等重点行业领域安全专项整治，加强对各类安全事故隐患的排查治理，健全重大隐患治理、重大危险源监控制度，完善预报、预警、预防和应急救援体系，坚决遏制重特大事故发生。加大食品安全专项整治力度，强化食品药品、餐饮卫生监管，保障人民群众的健康安全。深入推进社会诚信体系建设，整顿和规范市场经济秩序，优化金融生态环境，争创全国金融生态示范市。继续强化社会治安综合治理，严厉打击各种违法犯罪活动，不断提高动态环境下的治安管理和防范水平，确保社会大局稳定。

继续加强国防建设、民兵预备役和“双拥”工作，促进军政军民团结。全面贯彻党的民族宗教政策，发挥宗教界人士和信教群众在促进经济发展、构建社会和谐中的积极作用。充分发挥工会、共青团、妇联等人民团体的桥梁纽带作用。继续做好审计、统计、外事、侨务、对台、人防、气象、地震、档案、史志等工作。

五、坚持改革开放，为经济社会发展提供强大动力

今年是改革开放30周年，改革开放使许昌发生了历史性巨变。实现全面建设小康社会的宏伟目标，必须进一步解放思想，更新观念，开阔视野，以改革开放开创新的局面。

（一）创新发展机制，深化企事业单位改革

突出重点，规范操作，注重实效，又好又快地推进既定42家企事业单位改革改制。以引进战略投资者为重点，扎实做好许继集团、远航集团等企业的战略重组。按照政策，兼顾各方，积极推进文化、卫生、城建等事业单位的改革。完善已改制单位法人治理结构，健全内部运作机制，加强对股权流转的监管，不断焕发自身活力。加大破产、重组力度，从深层次上解决已改制企业历史遗留问题。鼓励和支持国内外大企业集团和非公有制经济向重点发展行业和区域集中，以存量引增量，以增量添活力、促发展。

（二）加快重点领域改革，不断增添发展活力

加快财税金融体制改革、农村综合改革等重点领域和关键环节改革步伐。深化财政体制改革，全面推进县级部门预算改革，进一步完善财政投资评审制度，逐步建立政府采购“管采分离”制度，打造民生财政、公共财政、阳光财政。建立完善政府债务预警机制，防范和化解财政风险。制定出台社保资金、住房公积金、土地收储资金、新农合资金监管办法，建立政府性资金长效监管机制。搭建政府投融资平台，实行政府投资项目代建制和回购制，着力破解制约城市发展的资金瓶颈。积极推进金融领域改革，完成农信社统一法人改革和专项票据兑付工作，推进市商业银行增资扩股。加快推进农村综合改革，全面推进集体林权制度改革，健全土地承包经营权流转机制，大力发展农民专业化合作组织。

（三）坚持开放带动战略，大力发展开放型经济

把“引进来”与“走出去”相结合，扩大开放领域，优化开放结构，提高开放质量，完善开放型经济体系。不断探索招商引资新方式，以园区和企业为载体，积极引进一批国内外知名企业。举办好花博会、药交会等节会活动，把花博会打造成为促进现代花木产业加快发展的高层次平台。大力推进企业上市，力争2－3家企业成功上市，上市后备队企业超过30家，引导已上市公司进行再融资。优化出口商品结构，在稳定发制品出口的同时，提高机电产品、纺织品、农副产品和高新技术产品的出口份额。鼓励有条件的企业到境外投资，发展对外工程承包和劳务合作。加强区域经济合作，承接产业转移，积极推进许昌与郑州的产业对接、空间对接，在郑许呼应发展中推进跨越发展。

六、坚持依法行政，努力建设服务型政府

以法治为上，以效能为先，以廉洁为本，着力打造服务型政府，让权力在阳光下运行，让人民赋予的权力始终为人民谋利益。

（一）在依法行政上提升新水平

全面推行依法行政，努力做到有权必有责、用权受监督、侵权要赔偿、违法要追究。建立健全决策权、执行权、监督权既相互制约又相互协调的运行机制，努力形成权责一致、分工合理、决策科学、执行顺畅、监督有力的行政管理体制。自觉接受人大及其常委会的法律监督、工作监督和政协的民主监督，严格执行人大及其常委会的决议、决定，广泛听取各民主党派、工商联和无党派人士的意见和建议。充分发挥监察、审计等职能部门的作用，强化政府层级监督。加强基层民主政治建设，组织好第六届村委会换届选举，做好选聘高校毕业生到村任职工作。强化行政执法监督，全面落实行政执法责任制。畅通行政复议渠道，办好行政复议案件。加强对公务员的法制教育和培训，进一步提高依法行政的意识和能力。

（二）在职能转变上迈出新步伐

在加强和改善经济调节和市场监管的同时，更加注重履行社会管理和公共服务职能。建立健全政府网站、市长信箱等多种反映群众诉求的渠道，了解民情、反映民意、集中民智，完善决策机制和利益协调机制。加快完善市、县（市、区）、乡、村4级为民服务快速通道，重点加强乡（镇）为民服务全程代理站的建设。巩固提高城市社区标准化建设成果，完善服务网络平台；积极推进农村社区建设，强化便民服务功能。切实加强对公用事业领域的监管。完善应急预案体系和管理体制，有效应对各类突发事件，提高危机管理和抗风险能力。

（三）在效能提升上实现新突破

深入开展“环境创优年”活动，积极营造良好的政策环境、服务环境、市场环境和法制环境。加快行政审批制度改革，市、县两级行政审批项目再精简10%，市级网上行政审批事项达到50%。逐步推进行政审批电子监察系统建设，使行政审批更加高效透明。加强电子政务建设，推进公共服务信息化，政务公开、村务公开普及率达到100%。深化机关标准化管理，市、县（市、区）两级标准化管理普及率达到100%。强化效能监察，完善快速联动的案件查处机制，加大对损害经济发展环境典型案件的查处力度。重点围绕与群众生产生活密切相关的领域抓好行风建设，坚决纠正涉及教育收费、医药购销、农民负担等领域的不正之风。

（四）在廉政建设上取得新成效

坚持艰苦奋斗，反对奢侈浪费，建设一支高素质的公务员队伍，致力形成清新、简约、责实、务本的良好政风；坚持权为民所用，情为民所系，利为民所谋，多做打基础、利长远、促发展、惠民众的事，致力形成求真务实的工作作风；坚持标本兼治、综合治理、惩防并举、注重预防的方针，致力形成教育、制度、监督并重的惩治和预防腐败体系。下大力气解决权力过分集中和缺乏制约的问题，规范土地和矿产资源开发、国有资产转让等公共资源管理。加大专项治理力度，重点解决环境保护、食品药品安全、安全生产、土地征收征用和房屋拆迁等方面群众反映强烈的问题。严肃查处各类违法违纪案件，深入开展治理商业贿赂，依法惩处腐败分子。

各位代表，2008年是实施“十一五”规划承上启下的关键一年，是我们站在新的历史起点上、向更高目标迈进的重要一年。让我们在中共许昌市委的正确领导下，认真贯彻党的十七大精神，全面落实科学发展观，解放思想，求真务实，锐意进取，开拓创新，为把许昌早日建设成为中原强市，提前实现全面建设小康社会的宏伟目标而努力奋斗！
